# Bridging Photoacoustic and Protoacoustic Imaging: Material Heterogeneity Effects on Proton Range Verification Using Time-of-Flight Analysis

**DOI:** 10.3390/bioengineering12101123

**Published:** 2025-10-20

**Authors:** Sangwoon Jeong, Wonjoong Cheon, Youngyih Han, Sungkoo Cho

**Affiliations:** 1 Department of Radiation Oncology, Wonju Severance Christian Hospital, Yonsei University Wonju College of Medicine, Wonju 26426, Republic of Korea; swjeong93@yonsei.ac.kr; 2 Department of Radiation Oncology, Seoul St. Mary’s Hospital, College of Medicine, The Catholic University of Korea, Seoul 06591, Republic of Korea; wjcheon@catholic.ac.kr; 3 Department of Radiation Oncology, Samsung Medical Center, Sungkyunkwan University School of Medicine, Seoul 06351, Republic of Korea; youngyih@skku.edu

**Keywords:** protoacoustics, proton therapy, proton range verification, acoustic heterogeneity, time-of-flight analysis

## Abstract

Photoacoustic and protoacoustic imaging share the common principle of acoustic wave generation through different excitation sources: optical absorption vs. proton Bragg-peak. These acoustic signals exhibit heterogeneity within the tissue, which strongly influence wave propagation and detection accuracy. In this study, we investigate how material variation affects time-of-flight (TOF)-based acoustic signal analysis in the context of protoacoustic proton range verification, providing insights relevant to broader photoacoustic imaging methodologies. A ±15 °C temperature difference in water caused only a 0.04 μs delay and thus had a minimal effect. In heterogeneous phantoms, lung-containing cases produced range errors up to 3.72 mm. In clinical scenarios, detectors aligned with air or low-density tissues showed large overestimations, up to 192.4 mm. Only 2 of 25 detector positions met the <2 mm error criterion. These results highlight that tissue composition and acoustic heterogeneity significantly influence protoacoustic wave propagation and range accuracy. Accurate range verification using protoacoustics must account for material variations along the wave path, particularly in lung regions, to ensure clinical applicability.

## 1. Introduction

Globally, the number of cancer patients continues to increase, with approximately 10 million dying of cancer in 2020 [1,2]. Radiation therapy is a method of treating cancer by irradiating tumor tissues while minimizing harm to surrounding healthy tissues. It is widely used in cancer treatment along with surgery and chemotherapy [3,4,5]. The representative radiation used in radiation therapy includes photons, electrons, and protons. Photons and protons are used when a tumor is deep within the body, whereas electrons are used for surface tumors.

Photons are utilized in deep tissue treatment because their energy gradually reduces as they penetrate an object. However, photons can provide unnecessary doses to organs at risk (OAR) along the path to the tumor. Various treatment techniques using photons have been studied to minimize unnecessary doses to the OAR; however, addressing the unique characteristics of photons remains a challenge [6,7]. Proton therapy, on the contrary, offers advantages over photon therapy. A proton beam loses energy when it interacts with an object, and delivers a high dose when it reaches a specific energy level. This peak is known as the Bragg-peak.

Bragg-peak characteristics significantly reduce unnecessary doses to the OAR [8,9]. However, if the Bragg-peak is positioned outside the target area, it can cause considerable damage to the surrounding OAR. Therefore, a precise treatment is crucial to ensure the accuracy of the proton beam range. Unlike a photon beam, that can be imaged after passing through the patient, a proton beam is fully absorbed within the tissue. Because no transmitted signal remains, the beam path cannot be visualized during treatment. This makes in-vivo range verification challenging. Additionally, it is challenging to confirm the proton beam range accuracy because of variations in the patient’s gastrointestinal system, respiration, heart movement, and anatomical structure [10]. Considering these factors, an appropriately sized margin is applied to the tumor, and treatment is conducted via irradiation with a proton beam [11]. Confirming the accuracy of the proton beam range can help to reduce the margin and minimize the risk to normal tissues.

Several methods have been developed to measure the proton beam range, including positron emission tomography (PET), prompt gamma radiation, and protoacoustic waves [12]. PET detects electron pairs of 511 keV emitted through β+ decay of neutron-deficient nuclei, occurring in a 180-degree range. At the Bragg-peak, β+ decay occurs through a nuclear fission reaction resulting from proton matter interactions. Electron pairs are generated over milliseconds to minutes, enabling confirmation of the Bragg-peak position. However, most of these emitters are created before the Bragg-peak rather than at the point of maximum energy deposition. As a result, the PET signal is weaker and slightly displaced from the true Bragg peak, limiting its accuracy for direct range verification. Moreover, the spatial resolution of detectors is limited [13]. Prompt gamma-rays are released during the inelastic interactions of protons with the nucleus of the tissue at the Bragg-peak. However, prompt gamma rays are generated isotopically, which lowers measurement efficiency when a slit-type collimator is used. In addition, background noise arises from the generation of secondary neutrons, which makes these methods challenging to use in treatment environments [14]. In contrast, the protoacoustic wave method allows for easy monitoring of the proton beam range during treatment because it permits miniaturization of the measuring equipment and provides real-time data.

Protoacoustic and photoacoustic waves share similar physical principles, both originating from thermoelastic expansion caused by energy deposition. However, protoacoustic waves are generated by the localized heating from proton beam energy at the Bragg-peak, while photoacoustic waves arise from optical photon absorption [15]. The characteristics of protoacoustic waves change according to the shape and width of the proton beam, whereas photoacoustic waves are influenced by tissue characteristics and structure [16]. Methods for measuring the position of a proton beam using protoacoustic waves include backpropagation, time reversal (TR) reconstruction, and time-of-flight (TOF). The 3D filtered back projection method is similar to CT image reconstruction, where several detectors are placed around the area where the protoacoustic wave is generated, and the detected signal is reconstructed to determine the position of occurrence [17]. The TR-based method reconstructs the protoacoustic wave pressure distribution and visualizes the proton dose distribution to determine its position [18]. The TOF method utilizes the arrival time of a protoacoustic wave, distance to the detector, and speed of the sound to determine the proton beam range [19]. Based on these methodological foundations, numerous studies have explored experimental and clinical applications of protoacoustic imaging for proton range verification.

Various approaches have been proposed to verify proton beam range using acoustic signals generated at the Bragg peak. Earlier studies explored signal enhancement with gold markers [20], noise suppression with deep learning algorithms [21], and the development of high-sensitivity detectors capable of measuring low-amplitude protoacoustic waves [22]. Proton beam characteristics were also examined through both simulations and experimental water-phantom measurements [16,23,24]. These efforts established the physical feasibility of acoustic-based proton range verification. More recently, several investigations have demonstrated clinically relevant applications: sub-millimeter range accuracy using pulsed proton beams and abdominal phantoms co-registered with ultrasound [25,26], single pulse range verification on a synchrocyclotron [24], and real-time 3D protoacoustic imaging with a 256-channel array [27]. Collectively, these advances indicate that protoacoustic imaging has reached a stage of technical maturity and translational feasibility.

Despite these advancements, most prior investigations have focused on homogeneous or water-equivalent media, leaving the effects of acoustic heterogeneity largely unexplored. In particular, the spatial variation in tissue composition derived from clinical CT data manifested as local differences in Hounsfield units (HU) can substantially influence the speed of sound and wave propagation, potentially compromising range estimation accuracy.

In this study, we investigated the impact of tissue property variations (HU standard deviations) on protoacoustic wave propagation by comparing simulations using uniform (zero HU variation) and non-uniform (with HU variation) phantom materials, including water and combinations of soft tissue and bone. Furthermore, we assessed various detector positions across five clinical cases, identifying optimal detector placements that minimize interference with treatment plans and maximize measurement accuracy. By addressing the influence of HU variations on protoacoustic wave accuracy, this study provides insights to enhance the precision and reliability of protoacoustic-based proton range verification in clinical practice.

## 2. Materials and Methods

### 2.1. Background

Protoacoustic waves are generated by rapid thermoelastic expansion following the energy deposition of a pulsed proton beam. As the proton beam transfers kinetic energy into tissue, localized thermal energy is generated through electromagnetic interactions, which in turn causes an increase in pressure. This pressure rise results in the emission of acoustic waves, referred to as protoacoustic or thermoacoustic waves. The initial pressure distribution $P_{0}(r)$ induced by the proton beam under thermoelastic conditions can be described by:

(1) $$P_{0}(r)=\Gamma \left(r\right)\rho \left(r\right)Dose\left(r\right)$$

where Γ(r) is the Gruneisen coefficient which represents the efficiency of converting deposited thermal energy into pressure. It is defined as:

(2) $$\Gamma (r)=c^{2}(r)\beta (r)/Cp(r)$$

where $c\left(r\right)$ is the speed of sound [m/s], $\beta \left(r\right)$ is the thermal expansion coefficient $\left[k^{-1}\right]$, and $Cp\left(r\right)$ is the specific heat capacity [J/(K·kg)], all of which vary spatially depending on the tissue type. ρ(r) [$kg/m^{3}$] denotes the local mass density of the material. These spatially varying parameters were derived from CT HU values using publicly available biological tissue property databases [18].

Acoustic attenuation during wave propagation was modeled using a frequency power-law, expressed as:

(3) $$\alpha (\omega )=\alpha_{0}\omega^{d}$$

where $\alpha_{0\;}[Np/((rad/s)\cdot m)]$ is the material-dependent attenuation pre-factor, and d is the power-law exponent. In this study, d was set to 1.05, a value commonly used for soft tissues [28]. As reported in previous studies [15] the speed of sound varies with frequency by less than 0.3% in soft tissues such as liver and fat at 1 MHz, but can increase by up to 4.3% in high-contrast media like bone and lung at lower frequencies (e.g., 50 kHz). The temporal profile of the proton pulse was modeled as a Gaussian function G(t), and the resulting time-dependent initial pressure distribution was defined as:

(4) $$P_{0}(r,t)=P_{0}(r)⊗G(t)$$

where ⊗ denotes convolution over time. This accounts for the temporal broadening of the pressure signal due to the pulse duration of the proton beam. Equations (1)–(4) are derived from the thermoelastic model of photoacoustic wave generation and acoustic propagation in [29,30,31,32].

The propagation of protoacoustic waves was simulated using the k-Wave MATLAB toolbox (version 1.4, MathWorks, Natick, MA, USA), which solves the full acoustic wave equation in heterogeneous media [33]. Simulations were performed using voxel-wise distributions of speed of sound and density, which were computed by mapping CT HU values to material properties according to previously validated models [34].

### 2.2. Three Types of Analysis

This study evaluated the impact of tissue property variations specifically, variations in CT-derived sound speed and density on the accuracy of proton range measurements using protoacoustic waves, as well as the effect of detector positioning based on clinical CT data. First, we compared protoacoustic wave arrival times in a water phantom under uniform and non-uniform material property conditions. Next, phantoms were constructed using soft tissue, lung, and bone segments extracted from an ATOM^®^ phantom (CIRS tissue simulation and phantom technology, Asbury Ave, Norfolk, VA, USA), and we evaluated how both the presence of tissue property variation and the location of the Bragg-peak affected proton range estimation. Finally, we assessed the feasibility of accurate proton range measurement using human CT scans in five representative treatment cases and identified optimal detector positions based on protoacoustic signal behavior.

#### 2.2.1. Impact of Spatial Temperature Variation in Water Phantom

In clinical settings, localized heating due to proton beam irradiation may lead to non-uniform temperature distributions within the medium, even if the overall average temperature remains unchanged. Local temperature increase causes a corresponding increase in the sound velocity and a slight decrease in density due to thermal expansion, thereby altering the local acoustic impedance and the phase of propagating waves. To evaluate this effect, we constructed water phantoms with spatial temperature variations having standard deviations of ±5 °C, ±10 °C, ±15 °C while maintaining a uniform average temperature of 25 °C. At the reference temperature, the speed of sound and density of water were set to 1498 m/s and 997 $kg/m^{3}$, respectively. The corresponding spatial fluctuations in sound speed and density were applied using temperature-dependent conversion models. This approach allowed us to isolate the impact of intra-tissue thermal heterogeneity on protoacoustic wave arrival time and proton range estimation accuracy, independent of changes in the bulk medium properties.

#### 2.2.2. Effects of Tissue Composition and Property Uniformity

We assessed how variations in tissue properties and material composition affect the accuracy of proton range estimation using protoacoustic wave arrival times. Mean and standard deviation values of HU for soft tissue, bone, and lung were extracted from the ATOM^®^ phantom CT images. These values were used to generate voxel-wise acoustic property maps for each tissue type, including speed of sound and density, as listed in Table 1 [34]. The mean HU and standard deviation values were consistent with those reported in previous CT-based studies [18,19], supporting their suitability for simulation of realistic tissue properties.

First, we constructed homogeneous phantoms consisting of a single tissue type soft tissue, bone, or lung. For each tissue, two versions were created: one with uniform material properties (zero HU variation) and another with non-uniform material properties derived from clinical HU distributions. These were referred to as the uniform and non-uniform phantoms, respectively.

**Figure 1:**
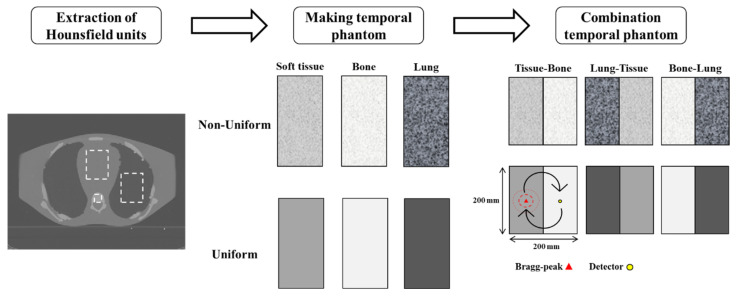
Workflow for generating a heterogeneous water phantom with spatially varying acoustic properties based on CT-derived tissue composition and temperature distribution.

Next, to assess the combined effects of tissue composition and property variation, heterogeneous phantoms were generated by combining two tissue types in equal proportions: soft tissue-bone, soft tissue-lung, and bone-lung. Each combination was simulated under both uniform and non-uniform conditions, resulting in a total of 12 heterogeneous phantoms (6 uniform and 6 non-uniform). As shown in Figure 1, all phantoms in each material combination had the same average acoustic properties; thus, any difference in results arises solely from the presence or absence of intra-tissue variation.

#### 2.2.3. Appropriate Detector Positioning Based on Clinical CT Cases

**Figure 2:**
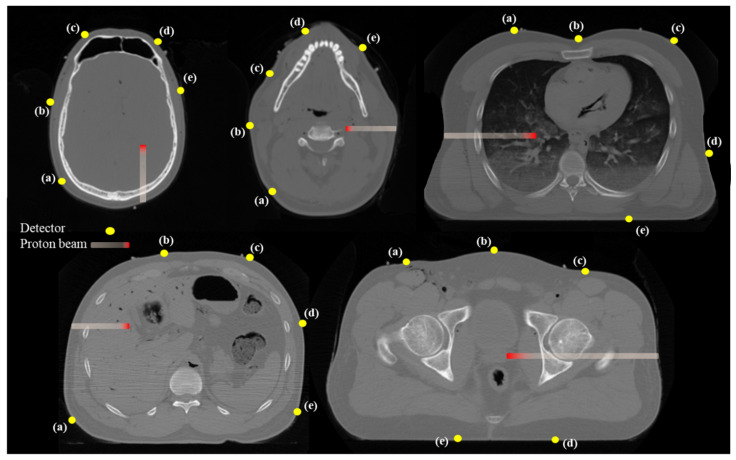
Axial CT images showing the beam path (red) and detector positions (yellow dots) for five clinical sites used to evaluate detector placement adequacy. Each letter (a–e) indicates the position of the detector. Detectors were placed around each target in positions that did not interfere with the proton beam path to avoid perturbation of dose delivery while ensuring adequate acoustic signal reception.

To determine optimal detector locations for protoacoustic wave acquisition in real CT images, we conducted simulations using human CT data from five representative clinical cases: brain, head and neck (H&N), liver, prostate, lung. The male whole-body CT dataset was obtained from the publicly available University of Iowa repository [35]. Tumor locations were defined as follows: the left occipital lobe (brain), left side of the cervical spine (H&N), central liver region, left posterior prostate, right lobe lung (Figure 2). In each case, five different candidate detector positions were evaluated to account for anatomical variability and practical placement feasibility. While detector positioning on the posterior or anterior surface (e.g., back or abdomen) may cause discomfort depending on patient posture, the primary focus of this study was to assess how internal tissue composition along the signal path affects the accuracy of proton range estimation.

For consistency across cases, a single high-energy Bragg-peak beam was simulated using the Monte Carlo platform TOPAS. The Bragg-peak position was determined by defining a target region within the CT and placing the beam such that its maximum energy deposition occurred at the target center. The protoacoustic wave arrival time at each detector was then simulated. The average HU value along the straight-line path from the Bragg-peak to the detector was converted to the corresponding speed of sound, enabling TOF-based range calculation. A protoacoustic signal was considered valid if the peak-to-valley amplitude exceeded 0.2 mPa. The most appropriate detector position was determined by comparing the TOF-based calculated range to the known actual range, minimizing the range error.

### 2.3. Simulation Parameter

All simulations were performed in a three-dimensional environment using the k-Wave MATLAB toolbox. The spatial resolution was set to an isotropic voxel size of 1 mm (pixel size and slice thickness), and the temporal resolution was 10 ns per time step. The total simulation time ranged from 100 to 400 μs, depending on the detector location. For the water phantom and heterogeneous tissue simulations (Section 2.2.1 and Section 2.2.2), a cubic volume of 200 mm × 200 mm × 200 mm was modeled, with the Bragg-peak and detector positioned 100 mm from the phantom center. For the human CT-based simulations (Section 2.2.3), clinical image data with a matrix size of 512 × 512 × 21 voxels was used, and both the Bragg-peak and detector were located on the central axial slice (11th slice).

In clinical proton therapy, beam energies typically range from 84 to 162 MeV. At these energy levels, a pulse of approximately 1 × $10^{7}$ protons can deliver about 1.0 cGy of dose at the Bragg-peak in water-equivalent tissue. According to thermoelastic theory, this dose would generate an initial pressure of approximately 1.2 Pa in soft tissue. To ensure numerical stability and prevent non-physical nonlinear effects during simulation, we scaled the peak pressure amplitude to 12 mPa. This value was used for Bragg-peak sources, while a lower pressure of 2 mPa was applied at multiple locations along the entrance path of the proton beam to simulate continuous energy deposition. The same Gaussian-shaped pulse waveform (10 μs total width) was used for both source types. To identify the Bragg-peak signal, we selected the global maximum of the detected acoustic waveform, based on the assumption that the Bragg-peak generates the highest pressure due to the concentrated energy deposition.

Acoustic wave propagation was modeled in heterogeneous media by assigning spatially varying speed of sound and mass density values to each voxel. These properties were derived from the CT Hounsfield units using previously validated tissue property conversion models [34]. Frequency-dependent acoustic attenuation was also incorporated using a power-law model with a spatially varying attenuation coefficient ${(\alpha }_{0})$ based on tissue type, and a fixed exponent (d=1.05) applied throughout the domain. To prevent boundary reflections and ensure realistic wave propagation, perfectly matched layers (PMLs) were applied at the simulation boundaries using a thickness of 20 grid points, an absorption coefficient of 2.0, and external PML positioning. All simulations were accelerated using GPU-based computation with single-precision arithmetic.

### 2.4. Evaluation

The accuracy of proton range estimation was evaluated using the time-of-flight method. In this approach, the arrival time of the protoacoustic wave at the detector is used to estimate the distance between the Bragg-peak and the detector. The estimated range ($D_{TOF}$) was calculated using the following equation:

(5) $$D_{TOF}=T_{peak}\cdot c$$

where $T_{peak}$ is the time at which the peak protoacoustic signal is detected, and c is the average speed of sound along the path between the detector and Bragg-peak. For each simulation case, the true distance ($D_{real}$) was known from the phantom geometry.

To assess the accuracy of the method, the absolute distance error (∆D) and relative error ($\varepsilon_{D}$) were calculated.

(6) $$∆D=|D_{real}-D_{TOF}|$$

(7) $$\varepsilon_{D}=∆D/D_{real}\cdot 100$$

Equations (5)–(7) follow standard TOF formulations commonly used in ionoacoustic range verification to derive the estimated distance and evaluate absolute and relative errors [23,25,36,37].

In the homogeneous and heterogeneous phantom simulations (Section 2.2.2), the influence of intra-tissue acoustic property variation on range estimation was assessed by comparing TOF-derived distances obtained from phantoms with uniform and non-uniform material properties. This allowed the effect of HU-derived spatial heterogeneity on proton range estimation to be quantitatively evaluated. In the clinical CT-based cases (Section 2.2.3), the detector position was varied to identify locations that minimize range estimation error. No correction was applied for pulse width-induced delay, as a consistent pulse shape was used across all cases. Signal quality was assessed qualitatively, and no filtering or denoising was applied.

## 3. Results

### 3.1. Impact of Temperature Deviation in Water Phantom

**Figure 3:**
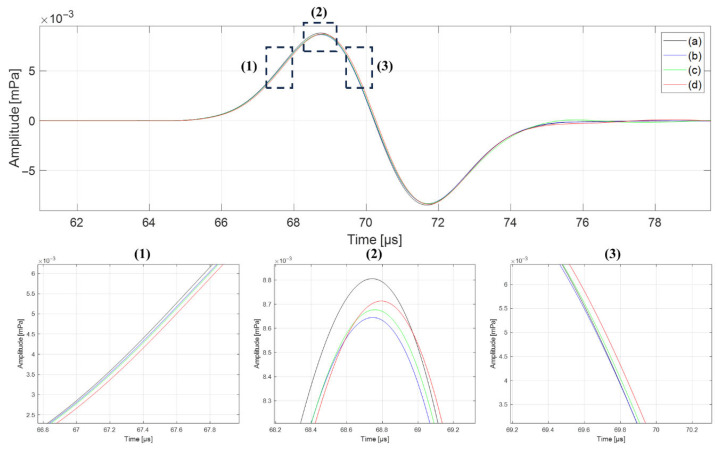
Protoacoustic waveforms under spatial temperature deviations of (**a**) 0 °C, (**b**) ±5 °C, (**c**) ±10 °C, and (**d**) ±15 °C in a water phantom. Insets show differences in wavefront rise, peak, and decay timing.

The effect of spatial temperature variation on protoacoustic wave arrival time was evaluated using water phantoms with increasing thermal standard deviations. For temperature deviations of 0 °C, ±5 °C, ±10 °C, and ±15 °C, the measured arrival times of the protoacoustic wave were 68.74 μs, 68.75 μs, 68.76 μs, and 68.79 μs, respectively (Figure 3).

### 3.2. Effects of Tissue Composition and Property Uniformity

**Figure 4:**
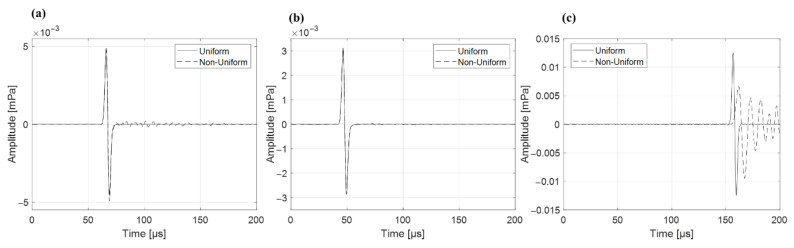
Comparison of protoacoustic waveforms obtained from homogeneous phantoms composed of (**a**) soft tissue, (**b**) bone, and (**c**) lung. Solid and dashed lines represent simulations with uniform and non-uniform acoustic properties, respectively.

To evaluate the impact of intra-tissue acoustic property variation on proton range estimation, protoacoustic simulations were performed using homogeneous phantoms consisting of soft tissue, bone, or lung. For soft tissue, the estimated Bragg-peak distances were 103.1 mm under uniform and 103.4 mm under non-uniform conditions. Bone showed identical values of 100.2 mm in both cases. In the lung, the estimated distance increased from 102.3 mm (uniform) to 105.6 mm (non-uniform), resulting in a 3.3 mm difference (Figure 4).

In the heterogeneous phantom simulations, six material combinations were evaluated with two Bragg-peak placements per pair, resulting in twelve configurations. Across all cases, the estimated distances from non-uniform phantoms were consistently larger than those from the corresponding uniform phantoms, regardless of Bragg-peak location. The lung-bone configuration showed increases of 3.62 mm and 3.72 mm. Tissue-lung phantoms showed increases of 2.87 mm and 2.95 mm. Bone-tissue phantoms showed smaller differences of 0.15 mm and 0.17 mm (Table 2).

### 3.3. Appropriate Detector Positioning Based on Clinical CT Cases

**Figure 5:**
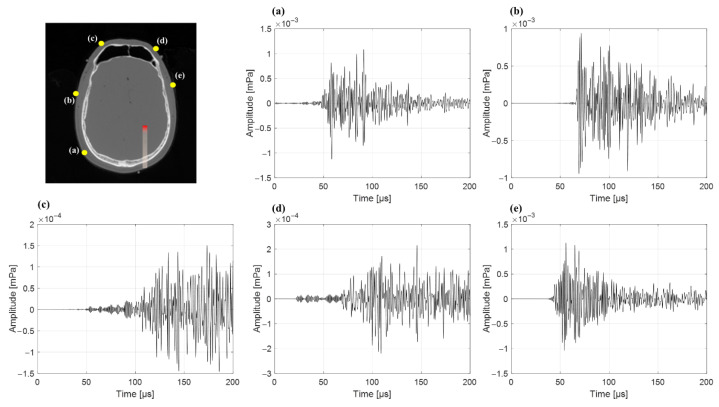
Protoacoustic signals detected at five different sensor positions (**a**–**e**) in the brain treatment case. The left image shows the detector arrangement (yellow dots) and proton beam path (red line) in the brain CT slice. The corresponding waveforms on the right represent the acoustic pressure signals recorded at each detector position.

In the five clinical CT-based cases, the protoacoustic range estimated by TOF-based calculation ($D_{TOF}$) was compared with the known Bragg-peak position ($D_{real}$) across five different detector positions per case. The detailed results for each treatment site and detector location are summarized in Table 3. Overall, the accuracy of range estimation varied depending on the sensor location. In the brain case, the ∆D smallest was 0.73 mm (position a), while the largest was 108.12 mm (position c). In the H&N case, position (b) yielded the most accurate result (∆D = 13.74 mm, $\varepsilon_{D}$ = 13.88%) (Figure 5). The liver case showed relatively small error at position (d) with ∆D = 17.60 mm, compared to much larger discrepancies at other positions. In the prostate case, position (b) again provided the most accurate estimation with ∆D = 3.39 mm. For the lung, all detector positions exhibited large deviations, with the smallest error at position (b), yet still reaching ∆D = 31.59 mm.

## 4. Discussion

### 4.1. Validation in Homogeneous Phantom

Protoacoustic range verification is typically validated using water phantoms due to their homogeneous and well-characterized acoustic properties. In this study, spatial temperature deviations up to ±15 °C were introduced while maintaining a consistent mean temperature to evaluate the effect of thermal non-uniformity. The resulting protoacoustic signal exhibited a maximum delay of 0.04 μs, corresponding to a distance error of less than 0.1 mm. This level of deviation is negligible in the context of clinical proton range verification. Previous studies [38,39] reported no measurable discrepancy between experimental and simulated protoacoustic waveforms in water, supporting the conclusion that moderate intra-medium temperature heterogeneity does not significantly impact range estimation. These results confirm that water phantoms remain a robust and reliable medium for protoacoustic validation, even under non-uniform temperature conditions.

Protoacoustic-based proton range verification must ultimately be applicable within the human body, where tissues exhibit varying acoustic properties. To investigate this, we constructed both uniform and non-uniform phantoms using three representative tissues: soft tissue, bone, and lung. In the homogeneous phantom simulations, the calculated Bragg-peak distances differed by less than 1 mm between the uniform and non-uniform phantoms for soft tissue and bone. However, in the lung phantom, which had the lowest density and sound speed among the three, the non-uniform case yielded a 3.3 mm longer estimated range. This indicates that tissue property variation has a greater impact in low-density media such as lung. In [40], have demonstrated excellent agreement between simulations and experiments in homogeneous materials like polyethylene, supporting the fundamental accuracy of protoacoustic TOF methods under uniform conditions. However, these studies did not explore the effects of spatial heterogeneity in acoustic properties, which are prevalent in actual human tissues. By incorporating clinically derived HU variations into our simulations, we revealed that protoacoustic range estimation becomes increasingly susceptible to error in acoustically low-density tissues. This discrepancy most pronounced in the lung phantom highlights the need to account for intra-tissue variation in order to avoid systematic overestimation of the Bragg-peak location. This step is essential for achieving robust and reliable protoacoustic range verification in practical treatment planning.

### 4.2. Influence of Tissue Heterogeneity on Range Accuracy

In all cases, the estimated Bragg-peak distances were consistently longer in non-uniform phantoms compared to their uniform counterparts. The largest discrepancies were observed in combinations involving lung tissue, which has the lowest density and acoustic impedance among the three materials. Specifically, the bone-lung combination exhibited range differences of 3.62–3.72 mm, and the lung-tissue combination showed differences of 2.87–2.95 mm. In contrast, the bone-soft tissue combination, which did not include lung, demonstrated minimal differences of 0.15–0.17 mm. The presence of lung within the acoustic path increases the sensitivity of protoacoustic TOF measurements to material property variations. This sensitivity likely arises from abrupt impedance transitions and heterogeneous sound speed distributions at interfaces between lung and denser tissues, which can distort wave propagation and lead to delayed signal arrival [41]. When lung is part of the beam path, protoacoustic-based proton range estimation should incorporate corrections for both intra-tissue heterogeneity and interfacial acoustic mismatches to maintain accuracy in clinical applications.

In the heterogeneous phantom simulations, although the actual geometric distance between the Bragg-peak and the detector was fixed at 100 mm, all configurations resulted in overestimated range values. The bone-lung phantom showed the largest deviation, with an estimated distance of up to 145.43 mm. The lung-soft tissue phantom exhibited a range of 125.78 mm, and even the tissue-bone phantom, which included only high-density materials, showed a modest overestimation of 104.82 mm. These discrepancies can be attributed to the cumulative effects of local acoustic heterogeneity and interfacial impedance mismatches. In phantoms containing lung tissue, abrupt changes in acoustic properties along the wave path lead to partial reflections, wavefront distortion, and group velocity dispersion, all of which contribute to delayed signal arrival times [42,43,44]. Moreover, the TOF method relies on an averaged speed of sound derived from CT-based HU values, which may not accurately capture local variations in complex tissue mixtures [19]. As a result, the calculated TOF translates into an extended distance, despite the physical geometry being constant. These findings highlight the need for advanced correction strategies or adaptive modeling approaches when applying protoacoustic range verification in anatomically heterogeneous regions.

### 4.3. Clinical Feasibility and Detector Placement Optimization

In the brain case, detectors (c) and (d) exhibited large range errors (∆D > 50 mm) due to the presence of the frontal sinus cavity between the Bragg-peak and the detector (Table 3). In the head-and-neck case, detector (a) showed the largest error (∆D = 84.86 mm), which can be attributed to thin air gaps along the acoustic path. In the liver case, detectors (b) and (c) produced significant errors due to gastrointestinal gas in the wave path. Notably, detector (b), despite being positioned closer to the Bragg-peak than detector (a), resulted in a larger range error of 26.68 mm. In the prostate case, detector (d) ($D_{real}$ = 130.38 mm) was located farther from the Bragg-peak than detector (e) ($D_{real}$ = 132.97 mm), yet the corresponding range errors were 11.34 mm and 41.72 mm, respectively, indicating that distance alone did not determine accuracy. The lung case showed the most substantial discrepancies, with ∆D values ranging from 31.59 mm to 192.39 mm across all detector positions. Similarly to the findings from the heterogeneous phantom simulations, these results highlight the significant influence of air-filled structures on protoacoustic wave propagation. Therefore, in clinical applications of protoacoustic-based proton range verification, the presence of air cavities, tissue composition along the beam path, and not just geometric distance must be carefully considered when selecting detector positions.

There are currently no established criteria for proton range errors in heterogeneous materials as part of quality assurance (QA) protocols. In clinical settings, water phantom-based QA typically allows a proton range deviation of less than 1 mm, and a spread-out Bragg-peak width tolerance of less than 2% or 2 mm, as recommended by AAPM TG-224 [45]. If these water-based QA thresholds are provisionally applied to heterogeneous conditions, only the brain case in this study partially satisfies the criteria, with 2 out of 5 detectors meeting the 2 mm range error requirement. All other anatomical sites (head and neck, liver, prostate, and lung) showed range errors exceeding these thresholds at all detector positions. To further improve clinical viability, advanced signal enhancement strategies and pre-treatment simulations that consider beam path tissue composition and acoustic heterogeneity should be incorporated. Additionally, integrating multiple detectors and using consensus-based range estimation may help reduce localized errors caused by anatomical irregularities.

Several studies have explored protoacoustic-based proton range verification using patient CT scans. However, these investigations have typically been limited to a small number of cases. For instance, evaluated range estimation by altering material composition in soft-tissue-based liver and prostate models, while [15] analyzed a prostate case that included bone structures. Reference [18] applied the TR method using detector arrays and reconstructed pressure distributions, which differs fundamentally from our TOF-based approach utilizing single-point detection. In contrast to prior work, our study systematically identified optimal detector positions across five distinct treatment scenarios using CT-derived anatomical models and TOF signal analysis.

### 4.4. Limitations and Future Perspectives

Previous research has also demonstrated the feasibility of protoacoustic range verification in homogeneous water phantoms using k-Wave simulations, with results validated through experimental measurements. Expanding upon this foundation, our study confirmed the applicability of protoacoustic signals in realistic, patient-based CT datasets, and further investigated the effects of both intra-tissue property variation and multi-material combinations on range estimation accuracy. Although the current study did not include experimental validation using anthropomorphic phantoms, the k-Wave framework employed has been widely adopted and shown to produce results consistent with physical measurements in prior studies. Future work will include experimental validation using human-equivalent phantoms to further confirm the clinical relevance of our findings.

## 5. Conclusions

In this study, we systematically evaluated the accuracy of protoacoustic-based proton range verification under varying acoustic conditions, from thermally varied water phantoms to heterogeneous tissue models and clinical CT datasets. The results demonstrated that tissue composition and acoustic heterogeneity, particularly in lung regions, can markedly influence TOF-based range estimates, while detector positioning also affects accuracy through both geometric and acoustic path factors. These findings confirm the feasibility of protoacoustic methods under realistic anatomical conditions and offer guidance for future clinical integration. By identifying how tissue-dependent variations alter propagation and signal arrival, this work establishes a framework for optimizing detector placement and improving range accuracy beyond conventional homogeneous calibration. Overall, the proposed approach enhances the clinical reliability of protoacoustic methods and provides a foundation for developing correction strategies and conducting experimental validation with anthropomorphic phantoms in future work.

## Figures and Tables

**Table 1:** CT-based Hounsfield units and corresponding tissue properties (density and speed of sound) used for uniform and non-uniform phantom construction.

	Hounsfield Units		Density [kg/m^3^]		Speed of Sound [m/s]	
	Mean	Standard Deviation	Mean	Standard Deviation	Mean	Standard Deviation
Soft-tissue	1042.5	62.5	1050.4	32.8	1567.1	36.8
Bone	1850.0	50.0	1574.4	19.3	2153.9	21.6
Lung	234.5	71.5	234.0	42.7	652.8	47.8

**Table 2:** TOF-based Bragg-peak distance estimates for uniform and non-uniform heterogeneous phantoms. Each tissue combination was tested with two Bragg-peak placements, and the difference in D_TOF values reflects the effect of intra-tissue acoustic heterogeneity.

Bragg-Peak	Detector	Type	TOF [μs]	$D_{TOF}$ [mm]	Difference [mm]
Bone	Lung	Uniform	101.05	141.81	+3.62
		Non-uniform	103.63	145.43	
Lung	Bone	Uniform	102.14	143.34	+3.72
		Non-uniform	104.79	147.06	
Bone	Tissue	Uniform	56.07	104.32	+0.15
		Non-uniform	56.15	104.47	
Tissue	Bone	Uniform	56.25	104.65	+0.17
		Non-uniform	56.34	104.82	
Lung	Tissue	Uniform	111.65	123.92	+2.95
		Non-uniform	114.31	126.87	
Tissue	Lung	Uniform	110.74	122.91	+2.87
		Non-uniform	113.33	125.78	

Difference [mm] = non-uniform $D_{TOF}\;-$ uniform $D_{TOF}$.

**Table 3:** Comparison of distances between proton range calculated by protoacoustic waves and real range in five treatment case.

Treatment Case		Detector Position				
		(a)	(b)	(c)	(d)	(e)
Brain	$D_{real}$ [mm]	94.7	111.14	141.03	116.5	74.65
	$D_{TOF}$ [mm]	93.97	112.97	249.15	171.678	91.19
	∆D [mm]	0.73	1.83	108.12	55.18	16.54
	$\varepsilon_{D}$ [%]	0.77	1.65	76.66	47.36	22.16
Head & Neck	$D_{real}$ [mm]	104.66	99.02	93.01	100.22	72.8
	$D_{TOF}$ [mm]	189.52	112.76	130.86	115.15	103.34
	∆D [mm]	84.86	13.74	37.85	14.93	30.54
	$\varepsilon_{D}$ [%]	81.08	13.88	40.69	14.90	41.95
Liver	$D_{real}$ [mm]	130.6	134.62	215.18	265.19	282.18
	$D_{TOF}$ [mm]	143.14	107.94	296.83	282.79	331.46
	∆D [mm]	12.54	26.68	81.65	17.60	49.28
	$\varepsilon_{D}$ [%]	9.60	19.82	37.94	6.64	17.46
Prostate	$D_{real}$ [mm]	163.03	121.02	127.26	132.97	130.38
	$D_{TOF}$ [mm]	202.09	117.63	90.35	121.63	172.1
	∆D [mm]	39.06	3.39	36.91	11.34	41.72
	$\varepsilon_{D}$ [%]	23.96	2.80	29.00	8.53	32.00
Lung	$D_{real}$ [mm]	130	143.69	241.05	252.56	182.22
	$D_{TOF}$ [mm]	95.71	175.28	322.37	444.95	245.62
	∆D [mm]	34.29	31.59	81.32	192.39	63.40
	$\varepsilon_{D}$ [%]	26.38	21.98	33.74	76.18	34.79

## Data Availability

The raw data supporting the conclusions of this article will be made available by the authors on request.
